# Supplementary material for: Recombinant Arginine Deiminase from Levilactobacillus brevis Inhibits the Growth of Stomach Cancer Cells, Possibly by Activating the Intrinsic Apoptosis Pathway
Source: Int J Mol Sci. 2024 Apr 9;25(8):4163. doi: 10.3390/ijms25084163 (PMC11050082; doi:10.3390/ijms25084163)
Supplement: Supplementary file 1 [file ijms-25-04163-s001.zip › ijms-2893537-Supplementary Materials.pdf]

Supplementary Materials

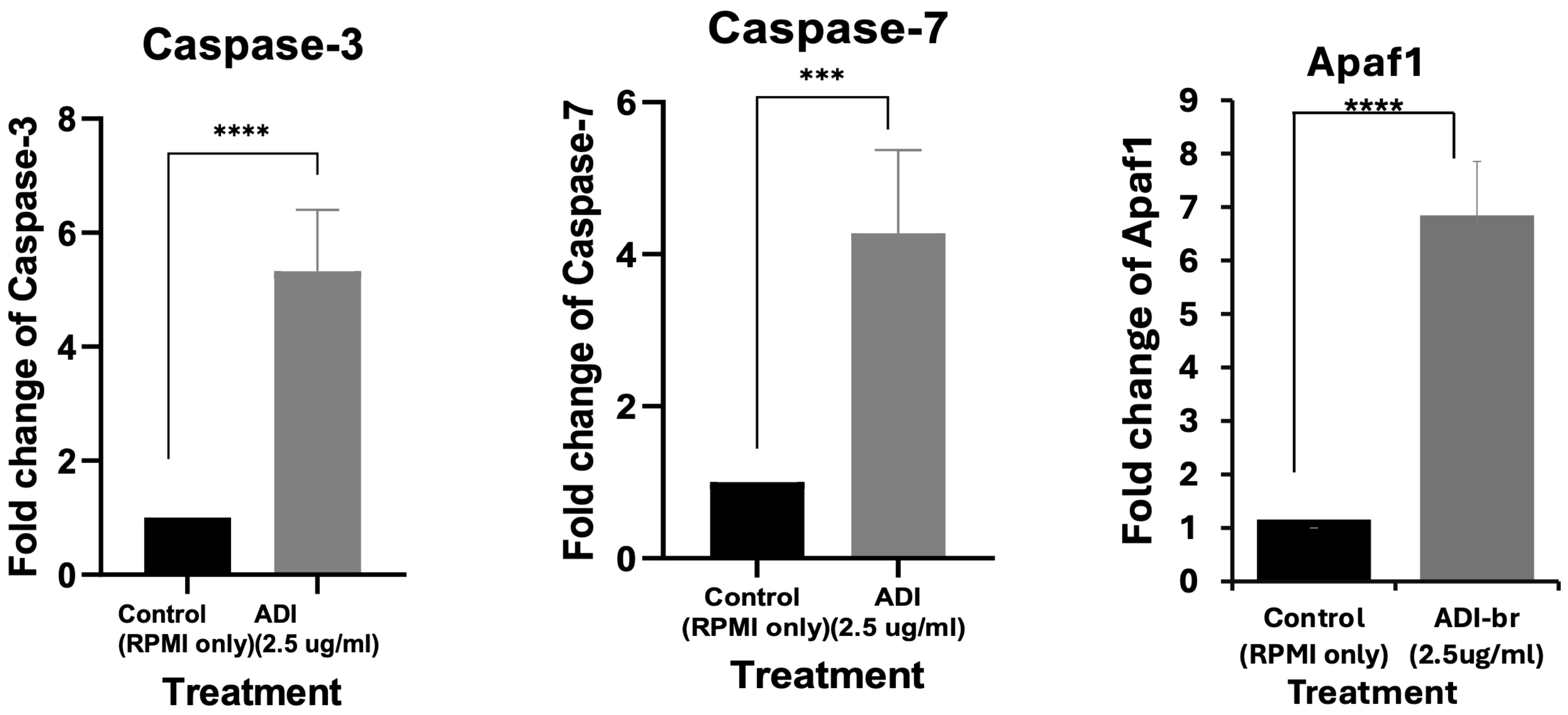

Figure S1. Preliminary RT-qPCR. Treatment of ADI_br (2.5 μg/ml) significantly upregulated the expressions (*caspase*-3, *caspase*-7, and *Apaf1*) of the intrinsic apoptosis-related genes. β2-microglobulin (*B2M*) was used as the internal control for the analysis following the *delta-delta* Cq method. Data presented are means of three replicate readings. Statistical analysis was conducted using one-way ANOVA implemented in GraphPad Prism 8.4.2. **** $p < 0.0001$, *** $p = 0.0002$.
